# Evaluation of the Key Advantages between Two Modalities of Boronophenylalanine Administration for Clinical Boron Neutron Capture Therapy Using an Animal Model

**DOI:** 10.3390/cells11172736

**Published:** 2022-09-01

**Authors:** Yu-Chuan Lin, Yi-Jang Lee, Yi-Wei Chen, Shan-Ying Wang, Fong-In Chou

**Affiliations:** 1 Nuclear Science and Technology Development Center, National Tsing Hua University, Hsinchu City 300044, Taiwan; 2 Department of Biomedical Imaging and Radiological Sciences, National Yang Ming Chiao Tung University, Taipei City 112304, Taiwan; 3 Department of Oncology, Taipei Veterans General Hospital, Taipei City 112201, Taiwan; 4 College of Medicine, National Yang Ming Chiao Tung University, Taipei City 112304, Taiwan; 5 Department of Medical Imaging and Radiological Technology, Yuanpei University of Medical Technology, Hsinchu City 300102, Taiwan; 6 Department of Nuclear Medicine, Far Eastern Memorial Hospital, New Taipei City 220216, Taiwan; 7 College of Nuclear Science, National Tsing Hua University, Hsinchu City 300044, Taiwan

**Keywords:** boron concentration, boron ratio, boron accumulation effect, pharmacodynamic, one step infusion, two step infusion, boronophenylalanine, boron neutron capture therapy

## Abstract

In clinical boron neutron capture therapy (BNCT), boronophenylalanine (BPA) administrations through one-step infusion (OSI) and two-step infusion (TSI) are the most widely used. This study compared the advantages of OSI and TSI using a human oral squamous cell carcinoma-bearing animal model. OSI was administered at a high-dose rate of 20 mg/kg/min for 20 min (total dose: 400 mg/kg) as the first step infusion. TSI was a prolonged infusion at a low-dose rate of 1.67 mg/kg/min for 15, 30, 45, and 60 min (total dose: 25, 50, 75, and 100 mg/kg) following the first step infusion. The sigmoid *E*_max_ model was used to evaluate the boron accumulation effect in the tumor. The advantages of TSI were observed to be greater than those of OSI. The observed advantages of TSI were as follows: a stable level of boron concentration in blood; tumor to blood boron ratio (T/B); tumor to muscle boron ratio (T/M); and skin to blood boron ratio (S/B). The boron accumulation effect in tumors increased to 68.98%. Thus, effective boron concentration in these tumor cells was achieved to enhance the lethal damage in BNCT treatment. Boron concentration in the blood was equal to that in the skin. Therefore, the equivalent dose was accurately estimated for the skin.

## 1. Introduction

Boron neutron capture therapy (BNCT) is a binary cancer treatment method based on a combination of ^10^B-containing drugs and thermal neutrons. Individual application of the ^10^B-containing drug and thermal neutron exhibit no therapeutic effect [1]. Boronophenylalanine (BPA) is a modified amino acid of the ^10^B-containing drug, a phenylalanine analog, widely used in clinical BNCT [2,3]. Several studies have suggested that BPA is transported into the cell through L-amino acid transporter 1 (LAT1) [4,5,6,7]. LAT1 overexpression is observed in various types of cancers and is related to the degree of malignancy and poor overall survival [8,9,10,11]. BPA selectively accumulates in the tumor to increase therapeutic effects while accumulating less in the normal tissues, thus reducing radiation damage. Therefore, a high concentration of ^10^B in the tumor is one of the indispensable factors for a successful BNCT.

In BNCT clinical trials, different methods of intravenous administration of BPA have been used. Patients with glioblastoma multiforme received the one-step infusion (OSI) at a high dose of 130–250 mg/kg BW of BPA for 2 h and is then irradiated with neutrons in two separate periods thereafter [12,13]. Since BPA in the blood continues to be eliminated after administration, the boron concentration in the blood must be monitored online by inductively coupled plasma atomic emission spectrometry (ICP-AES) or by prompt gamma detection [14,15]. The two-step infusion (TSI) method is a prolonged infusion at a low-dose rate of BPA after the first step infusion at a high-dose rate, and has also been used in BNCT clinical trials. In this method, the patient is administered BPA at a flow rate of 200 mg/kg/h for 2 h before neutron irradiation; then, the flow rate was reduced to 100 mg/kg/h in patients with head and neck cancer during neutron irradiation, ensuring that the boron concentration in the blood was maintained [16]. In Taiwan, patients receive TSI; the first-step BPA infusion at a high dose rate for 2 h and is followed by the second-step infusion at a low dose rate during neutron irradiation [17,18]. However, no studies have demonstrated the difference in pharmacodynamics (PD) between OSI and TSI during neutron irradiation.

Since the 1960s, the mathematical approach has been used to describe the concept of pharmacokinetic and pharmacodynamic (PK-PD) relationships between drug concentrations and effects [19,20,21]. The PK model evaluates the process of absorption and exclusion of drugs in the body. The PD model describes the time course of the pharmacological effects of drugs, which considers the mechanism of drug action and the main rate-limiting steps in the biology of the system [22]. The pharmacological target properties of a drug include binding to receptors, enzymes, transporters, ion channels, or DNA and inducing biological effects [23]. The typical PD models include fixed, linear, log-linear, *E*_max_ model, sigmoid *E*_max_ model, and indirect PD response [24,25]. However, the sigmoid *E*_max_ model explains the PD of drugs by Hill coefficients, determining the sigmoidicity or sensitivity between exposure and effects, which is known as the Hill equation [26]. The Hill equation has been used to evaluate the efficiency of amino acid delivery via transporters [27,28].

Individual administration of BPA induces no therapeutic effect in BNCT. The killing effects are induced by neutron irradiation when BPA targets the tumor via LAT1 transporters. The amount of ^10^B accumulated in the tumor is one of the keys to the success of BNCT treatment. BPA has rapid clearance in the blood after stopping infusion (OSI situation) [29]. In one patient, neutron irradiation was performed at this time [14]. In TSI, the second step infusion aims to maintain boron levels in the blood under neutron irradiation [14,15,16,17,18]. While the difference between OSI and TSI in terms of blood boron levels has been demonstrated, the accumulated effect of ^10^B in the tumor to achieve effective boron concentration in BNCT treatment between OSI and TSI are not clear during neutron irradiation. Therefore, in this study, OSI was a high-dose rate infusion of BPA as the first step infusion. TSI was a prolonged low-dose rate infusion after the first step infusion. The comparison of OSI and TSI in terms of BPA delivered to the tumor effects were assessed by the sigmoid *E*_max_ model, biodistribution, and boron ratios.

## 2. Materials and Methods

### 2.1. Cell Culture

The human oral squamous cell carcinoma cell line (SAS; JCRB) was maintained in Dulbecco’s modified Eagle’s medium/nutrient mixture F-12 (DMEM/F-12; GIBCO, Grand Island, NY, USA) containing 10% heat-inactivated fetal bovine serum (FBS), as well as 1% penicillin/streptomycin (100 U/100 μg/mL), in a humidified atmosphere of 5% CO_2_ and 95% air at a constant temperature of 37 °C.

### 2.2. Human Oral Squamous Carcinoma Cells in a Tumor-Bearing Mouse Model

BALB/c nude mice (male, 6-week-old) were purchased from BioLASCO Taiwan Co., Ltd., (Taipei, Taiwan). Before the experiment, the mice were kept in a cage for at least one week at 20–24 °C and with a humidity of 40–60%, with unlimited access to food and water. The mice were subcutaneously implanted with 1 × 10^6^ SAS cells suspended in 100 μL phosphate buffered saline (PBS) into the right forelimb of nude mice under anesthesia with 2–3% isoflurane. When the tumor volume was about ~70 mm^3^, the mice were used for the biodistribution study and PD analysis.

### 2.3. Biodistribution Study after OSI or TSI Administration

**Figure 1:**
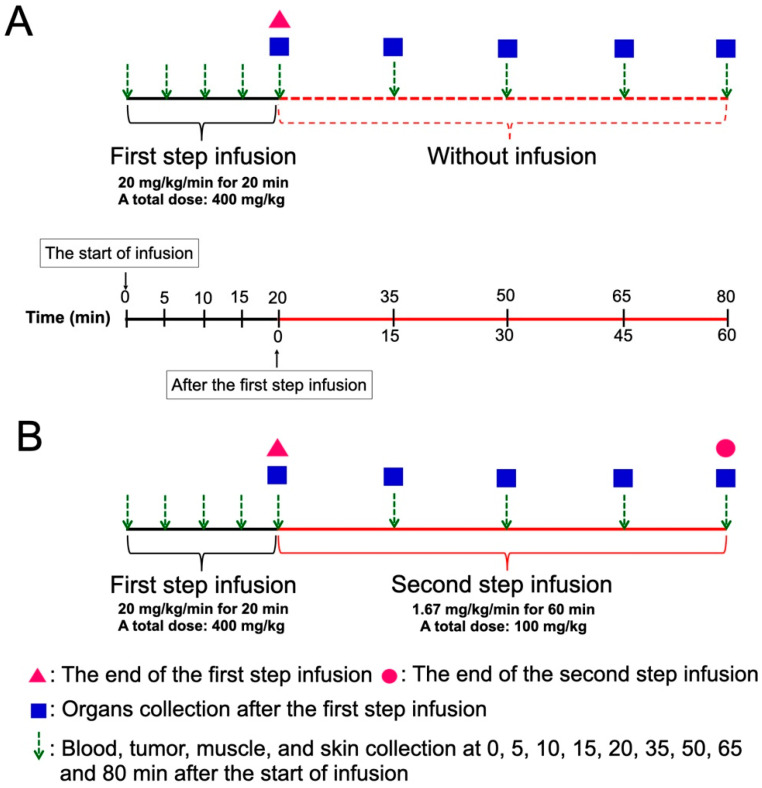
A scheme of the experimental design in (**A**) one-step infusion (OSI) and (**B**) two-step infusion (TSI) groups is shown. Total doses of the second step infusion at 15, 30, 45, and 60 min were 25, 50, 75, and 100 mg/kg, respectively.

Figure 1 shows the scheme of two methods of BPA administration. BPA was prepared as previously described [30]. The SAS tumor-bearing mice were randomly separated into OSI and TSI groups. For the first step infusion with a high dose rate of BPA, the mice were administered 20 mg/kg/min of BPA via the tail vein for 20 min (total dose: 400 mg/kg) by a microinjection pump. For the second step infusion at a low dose rate, 1.67 mg/kg/min of BPA was extendedly administered for 15, 30, 45, and 60 min (total dose: 25, 50, 75 and 100 mg/kg) after the first step infusion. In the OSI group, mice were administered a high dose-rate of BPA infusion for 20 min, at which point the infusion was stopped. In the TSI group, mice were administered with the first and second step infusion. Blood, tumor, muscle, and skin were collected at 0, 5, 10, 15, 20, 35, 50, 65, and 80 min after beginning the first-step infusion in the OSI and TSI groups. Mandible, tongue, heart, liver, pancreas, spleen, and kidney were collected at 0, 15, 30, 45, and 60 min after the first-step infusion in OSI and TSI groups. The tumor to blood boron ratio (T/B), tumor to muscle boron ratio (T/M), and skin to blood boron ratio (S/B) were evaluated.

### 2.4. Boron Concentration Analysis

The tissue samples were stored at –20 °C. Each sample was thawed at room temperature and weighed before digestion. Reagents comprising 3 mL of 65% nitric acid and 0.5 mL of hydrogen peroxide were used to digest the tissue samples in a microwave apparatus (MLS 1200 Milestone; Labtech SRL, Sorisole, Italy). For microwave digestion, samples were heated at 300 W for 3 min, followed by 250 W for 2 min, and cooled down for 20 min. The digestion solution was diluted with double-deionized water to a total volume of 25 mL. Boron concentrations in the samples were analyzed by ICP-AES (OPTIMA 2000 DV; PerkinElmer Instruments, Norwalk, CT, USA).

### 2.5. Analysis of Effects of Accumulated Boron in the Tumor

The effects of accumulated boron in the tumor were analyzed using a sigmoid *E*_max_ model, also called the Hill equation, which describes the relationship between the accumulation effect of boron and the boron concentration in the tumor. The Hill equation is as follows [26]:

$$E=\frac{E_{\max}\times C^{H}}{EC_{50}^{H}+C^{H}}$$

*E* is the predicted boron accumulation effect in the tumor to achieve effective boron concentration in BNCT treatment, *E*_max_ is the maximum effect in the tumor, C is the boron concentration in the tumor at a particular time, *EC*_50_ is the boron concentration in the tumor to reach 50% of the maximum effect, and *H* is the Hill coefficient. Herein, *EC*_50_ = 20, because the boron concentration in the tumor was at least 20 μg to achieve effective BNCT treatment [31]. In addition, *H* = 1, as BPA was noncooperatively transported into tumor cells without pre-loading other amino acids.

### 2.6. Curve Fitting and Statistical Analysis

Elimination of boron in blood after OSI was predicted through an exponential equation to fit the curve (exponential decay, single, and three parameters; SigmaPlot™, Systat Software, San Jose, CA, USA). The sigmoidal equation was used to predict a steady-state boron concentration in blood in the TSI group (sigmoidal curve, Hill equation, and three parameters; SigmaPlot™, Systat Software, San Jose, CA, USA). All data are shown as the mean ± standard deviation. The significant differences between OSI and TSI were analyzed using a t-test (Microsoft®, Excel®, Redmond, WA, USA); *p* ≤ 0.05 or *p* ≤ 0.01 was considered statistically significant.

## 3. Results

### 3.1. The Different BPA Accumulation and Boron Ratios between OSI and TSI Groups

**Figure 2:**
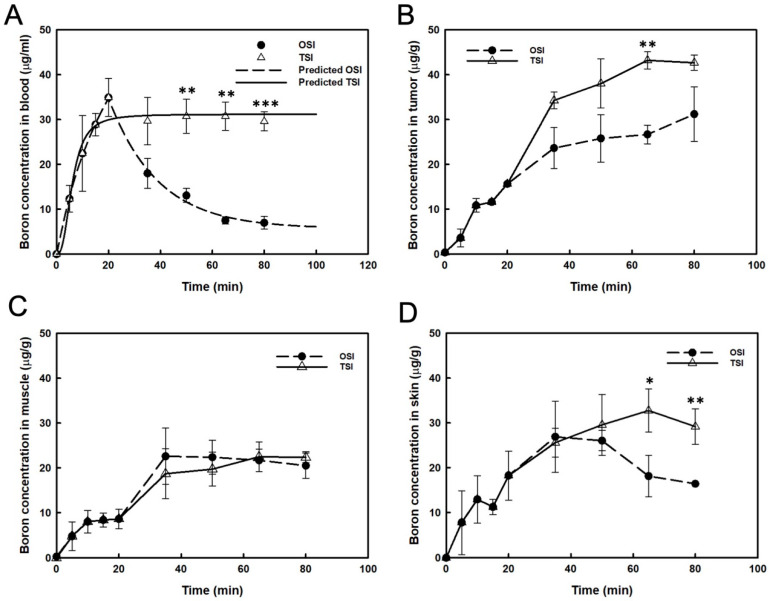
Boron concentrations were presented in (**A**) blood, (**B**) tumor, (**C**) muscle, and (**D**) skin. The predicted boron concentration in blood was analyzed by curve fitting and expressed as mean ± standard deviation. *: *p* ≤ 0.05, **: *p* ≤ 0.01, and ***: *p* ≤ 0.001 statistically significant difference, evaluated using paired *t*-test.

After the first step infusion in the OSI group, boron concentration in the blood decreased exponentially to 50% at 17 min (Figure 2A). The real boron concentration in the blood was highly correlated with the prediction in the OSI group (R^2^ = 0.994). In the TSI group, a steady-state of blood boron concentration was obtained, and the real value correlated well with the predicted value (R^2^ = 0.966; Figure 2A). A significant difference in blood boron concentration was observed between OSI and TSI groups after the first step infusion (Figure 2A). From 35 to 80 min, boron concentration in the tumor increased slightly after the beginning of infusion in the OSI group. In the TSI group, boron concentration in tumors was moderate from 65 to 80 min after the start of infusion. A significant difference between the OSI and TSI groups was observed 65 min after the start of infusion (Figure 2B). Boron concentration in the muscle was not significantly different between the OSI and TSI groups (Figure 2C). Boron concentration in the skin from 65 to 80 min in the TSI group was higher than that in the OSI group and differed significantly (Figure 2D). Table 1 shows the variance of boron ratios between the OSI and TSI groups. T/B ratio represented a time-dependent increase in the OSI group, which reached the highest value of 4.5. T/M and S/B were constantly maintained from 30 to 45 min and from 45 to 60 min, respectively, after the first step infusion in the OSI group. In the TSI group, T/B ratios showed stable levels for two different time intervals after the start of infusion. T/M and S/B ratios were constantly maintained from 30 to 60 min after the start of infusion. Boron ratios were constant in different time intervals in the TSI group.

### 3.2. The Difference in Boron Accumulation Effects in the Tumors in OSI and TSI Groups

**Figure 3:**
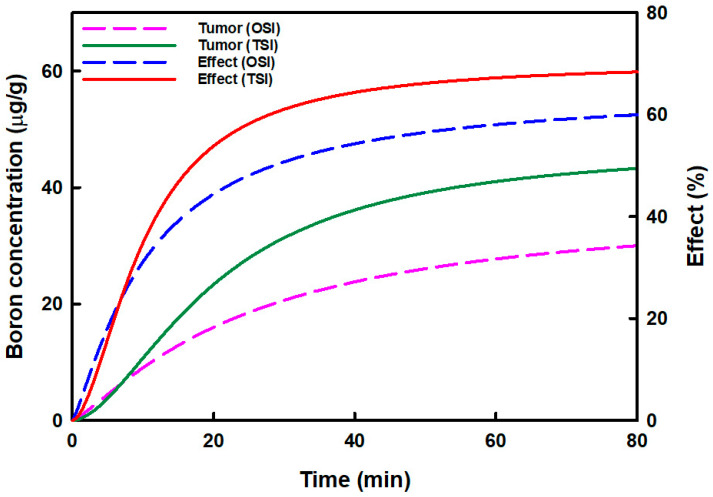
The relationship between the predicted boron concentration in the tumor and the boron accumulation effect in tumors in one-step infusion (OSI) and two-step infusion (TSI) groups is displayed. The predicted boron concentration in the tumor was analyzed by curve fitting, and the boron accumulation effect was analyzed by the sigmoid *E*_max_ model.

Figure 3 displays the relationship between the predicted boron concentration in tumors and boron accumulation effects in OSI and TSI groups. The predicted boron concentration in tumors and the boron accumulation effects increased more in the TSI group than in the OSI group. The highest boron concentrations in OSI and TSI groups were 31.46 μg/g and 44.48 μg/g, respectively. Boron concentration could be increased by 1.41-fold in the TSI group. The highest boron accumulation effects in OSI and TSI groups were 61.13% and 68.98%, respectively. Then boron accumulation effect in the TSI group increased 1.13-fold.

### 3.3. Biodistribution in Organs in OSI and TSI Groups

**Figure 4:**
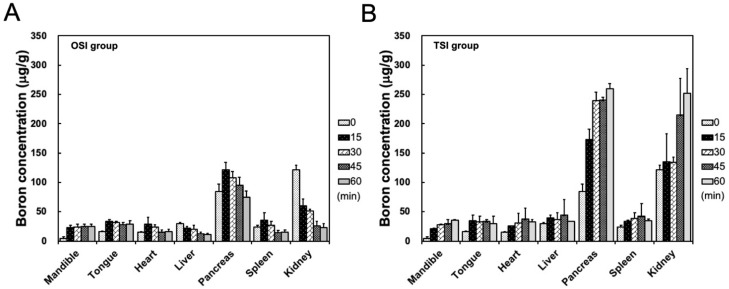
Biodistribution in organs was shown from 20 to 80 min in (**A**) one-step infusion (OSI) and (**B**) two-step infusion (TSI) groups. Data were expressed as the mean ± standard deviation.

After the start of infusion in the OSI group, boron concentrations in the mandible showed a stable level from 35 to 80 min. Boron concentration in the heart, liver, pancreas, spleen, and kidney decreased with time from 15 to 45 min after the start of infusion in the OSI group (Figure 4A). In the TSI group, boron concentration in the mandible exhibited a time-dependent increase within 1 h after the first step infusion (Figure 4B). In the TSI group, in addition to the pancreas and kidney, boron concentrations in other organs were slightly higher than in corresponding organs in the OSI group. High boron accumulations in the pancreas and kidney were observed in both the OSI and TSI group, especially in the TSI group (Figure 4).

## 4. Discussion

BPA targets the tumor, and then the tumor is irradiated with thermal neutrons to induce α and ^7^Li particles to deposit large amounts of energy, causing lethal damage to tumor cells [31]. Before BNCT is performed, tumor to normal tissue boron ratio (T/N) is determined by 4-borono-2-[^18^F]-fluoro-L-phenylalanine positron emission tomography in the patient [18]. During BNCT treatment, boron concentration in blood is assumed to be equal to that in normal tissue, based on T/N, and is then used to calculate the equivalent dose [17,18]. Therefore, when performing BNCT, boron concentration in the blood must be monitored. OSI and TSI are common BPA administration modalities in clinical BNCT [12,13,14,15,16,17,18].

Fukuda reported that BPA was quickly cleared from the blood after infusion was stopped [29]. In this study, boron concentration in the blood declined rapidly to 50% at 17 min after the first step infusion in the OSI group. In one study, for the modality of OSI administration in clinical BNCT, the patient was irradiated with two neutron periods [14]. To deliver an accurate equivalent dose to the tumor, the equivalent dose in the first period is compensated for by that in the second period. During the two periods of radiation, the irradiation position of the patient must be fixed. If the target regions of the patient shift during a long period of irradiation, an inaccurate equivalent dose would be delivered, and the treatment would be a failure. In this study, T/B ratios varied highly from 15 to 60 min after the first step infusion in the OSI group. This variance led to the calculation of incorrect equivalent dose.

The high infusion rate would shorten the time to reach a steady-state concentration compared to that achieved with a low infusion rate. The time to reach the steady-state was observed to be the same, but a higher steady-state drug level was obtained at a high infusion rate [32]. To maintain boron level in the blood during neutron irradiation, the patient was administered a second step infusion with low dose, and only a single neutron period was required in clinical BNCT [14,15,16,17,18]. In this study, the real boron concentration in blood reached the highest and then decreased to a constant level in TSI groups because of the decrease in infusion rate. This differed from the curve trend that predicted stable levels of boron in the blood. If the infusion rate was not decreased, the real boron concentration could be stable at a higher level. Overall, TSI could obtain a steady-state boron concentration in the blood, and in addition to stable levels of T/B, T/N, and S/B ratios. In this situation, an equivalent dose was accurately evaluated, and thus the therapeutic effects were enhanced in BNCT.

Joel et al. investigated the relationship between the total dose and infusion rate in boron concentration in the blood and tumor, and T/B ratios using a rat gliosarcoma model [33]. The results showed that the T/B ratio was independent of infusion time at 1 h after the end of infusion with the same infusion rate, but a different total BPA dose. At the same total BPA dose, boron concentrations in the tumor or blood at 1 h after a dose rate of 250 mg/kg/h infusion were similar to those after a dose rate of 125 mg/kg/h infusion [33]. The OSI administration was used in their study. In this study, the differences between OSI and TSI administration during neutron irradiation were investigated. Based on the 400 mg/kg of BPA administration in the first step infusion in both the OSI and TSI groups, the BPA dose was administered at 25, 50, 75, and 100 mg/kg in the second step infusion in the TSI group. This is because, in clinical BNCT, neutron irradiation was performed after the end of the final total BPA dose in the OSI administration, but low-dose BPA was continuously administered during neutron irradiation in the TSI administration to maintain boron concentration in the blood. T/B ratio was 4.5 at a dose rate of 20 mg/kg/min for 20 min (total BPA dose: 400 mg/kg) 1 h after the end of the first step infusion in the OSI group. To maintain boron concentration in the blood, a low dose infusion rate (1.67 mg/kg/min) was used for the TSI group. In the TSI group, T/B ratios were constant from 15 to 30 min and from 45 to 60 min after the second step infusion with the different total dose. Stable levels of T/M (1.9) and S/B (1.0–1.1) ratios were obtained 30 to 60 min after the second step infusion with the different total dose. The duration of infusion affects the drug distribution. After a prolonged infusion, a greater amount of the drug has entered the peripheral compartments (tumor and normal tissues) from the central compartment (blood). The concentration gradient between the central and peripheral compartments is reduced. Exponential decay will therefore decrease more slowly, with the initial decay predominantly due to elimination. In contrast, when the drug is continuously infused to achieve a steady state in the blood, the drug concentration between the central and peripheral compartments reaches equilibrium due to the infusion rate, to match elimination [34]. Hence, when BPA input was equal to output to reach a steady-state boron concentration in the blood, sufficient boron in the blood could be delivered to the tumor cells via LAT1 transporters in the TSI group. A stable level of boron ratios and the high boron accumulated in the tumor were obtained in the TSI group. After a short infusion, a small amount of the drug has diffused into the peripheral compartments from the central compartment because most drugs are eliminated from the central compartment. There is a high drug concentration gradient between the central and peripheral compartments [34]. Therefore, when BPA output was greater than input, boron concentration in the blood was exponentially decreased after BPA infusion in the OSI group. Boron was delivered to tumor cells in limited concentration. In clinical BNCT, the patient was continuously administered low-dose BPA after the end of high dose BPA in the first step infusion, to maintain boron concentration in the blood during neutron irradiation. The total equivalent dose in the tumor is based on normal tissue dose constraints. Therefore, the total dose of BPA is different for each patient in TSI administration due to the different irradiation time. If the OSI and TSI administrations were compared at the same total dose of BPA, the sampling time points were determined after the end of the total dose of BPA. At this time, boron concentration in the blood exponentially decreased in both the OSI and TSI groups. Hence, evaluation of the boron accumulation effect in the tumor and boron ratios in TSI administration during irradiation were not obtained.

The sigmoid *E*_max_ model could evaluate the relationship between drug concentration and receptors, enzymes, transporters, ion channels, or genetic material [23]. The commonly investigated drug binding receptor could produce therapeutic effects [24]. Shi et al. used the Hill equation to demonstrate that Na^+^ and Cl^−^ were co-transported with amino acids into the cell via amino acid transporters [28]. In this study, the relationship of BPA and LAT1 transporters to achieving effective BNCT treatment was evaluated using the sigmoid *E*_max_ model that defines PD in OSI and TSI groups, the so-called boron accumulation effect. The boron accumulation effect in the tumors in the TSI group was increased to 68.98% higher than that in the OSI group. The effective boron concentration in the tumor was more than 20 μg/g, resulting in successful BNCT therapy [31]. BPA is heterogeneously microdistributed in the tumor depending on the tumor size and characteristics [35]. The decrease in BPA uptake in tumors was caused by cell cycle distribution (G1 phase), quiescent cells, hypoxia, and necrosis [36,37,38]. In contrast, active tumor cells including G2/M phase cells and normoxia could uptake more BPA. Therefore, a modality of TSI administration could promote 61.12% to 68.98% of active tumor cells to achieve effective boron concentration in BNCT treatment. The TSI administration could potentially change the microdistribution in the tumor. An *E*_max_ of 100% in the boron accumulation effect was difficult to achieve due to the heterogeneous distribution of BPA in the tumor.

More than 95% of patients receive acute radiation-induced skin toxicity, which is called radiation dermatitis [39]. Boron concentration in the blood was used to estimate the equivalent dose of the skin in BNCT. Boron concentration in the blood is not equal to that in the skin, unless the S/B ratio is 1. Thus, evaluation of the S/B ratio is important for BNCT. In addition to boron concentration and S/B ratio, the compound biological effectiveness (CBE) was also considered to calculate the equivalent dose in BNCT. In one study, the CBE of the skin was 2.5, which was higher than in other normal tissue (1.3) [40]. Radiation damage to the skin was observed in BNCT [29]. In melanoma patients, S/B ratios were relatively constant at 1.31 ± 0.22 within 6 h after the end of BPA administration (OSI) [41,42]. In this study, the S/B ratio was 2.4 in the OSI group, and was constant from 45 to 60 min after the first step infusion. Generally, relative boron concentration in the blood is assumed to be equal to that in normal tissue because blood is easily obtained and analyzed in clinical BNCT. In the OSI group, boron concentration in the blood was used to calculate skin dose, which resulted in an underestimated equivalent dose, and could obviously induce radiation damage to the skin. In the TSI group, the S/B ratio was about 1, with an equal boron concentration in the blood and the skin. However, the accurate equivalent dose in the skin was estimated in the TSI group because of the equal boron concentration in the blood and the skin.

## 5. Conclusions

The OSI administration modality required more attention than TSI administration. Although T/B ratios in the OSI group were higher than in the TSI group, the T/M ratio was lower. Nevertheless, the advantages of TSI were greater than those of OSI administration. In the TSI group, stable levels of boron concentrations in the blood, T/B, T/M, and S/B were observed. In the TSI group, the predicted boron concentration in tumors increased from 31.46 to 44.48 μg/g, and the boron accumulation effect increased to 68.98%. Thus, in these tumor cells, effective boron concentration was achieved to improve the lethal effects of BNCT. The accurate equivalent dose in the skin could be estimated because boron concentration in the blood was equal to that in the skin. Boron concentration in other normal tissues in the TSI group was greater than in the corresponding organs in the OSI group, especially the pancreas and kidney. Thus, equivalent dose in normal tissues was carefully evaluated in the animal model.

## Figures and Tables

**Table 1:** Difference in boron ratios between one-step infusion (OSI) and two-step infusion (TSI) at sampling time points after the first step infusion.

Items		Time (min)			
		15	30	45	60
OSI	T/B	1.3	2.0	3.6	4.5
	T/M	1	1.2	1.2	1.5
	S/B	1.5	2	2.4	2.4
TSI	T/B	1.2	1.2	1.4	1.4
	T/M	1.8	1.9	1.9	1.9
	S/B	0.8	1.0	1.1	1.0

Notes: T/B: tumor to blood boron ratio, T/M: tumor to muscle boron ratio, S/B: skin to blood boron ratio.

## Data Availability

No applicable.
